# Supplementary material for: Seminoma and Embryonal Carcinoma Footprints Identified by Analysis of Integrated Genome-Wide Epigenetic and Expression Profiles of Germ Cell Cancer Cell Lines
Source: PLoS One. 2014 Jun 2;9(6):e98330. doi: 10.1371/journal.pone.0098330 (PMC4041891; doi:10.1371/journal.pone.0098330)
Supplement: File S1 — ZIP file containing DMRforPairs output for significant regions. Please start from the html files. (ZIP) [file pone.0098330.s008.zip › figures/34398.pdf]

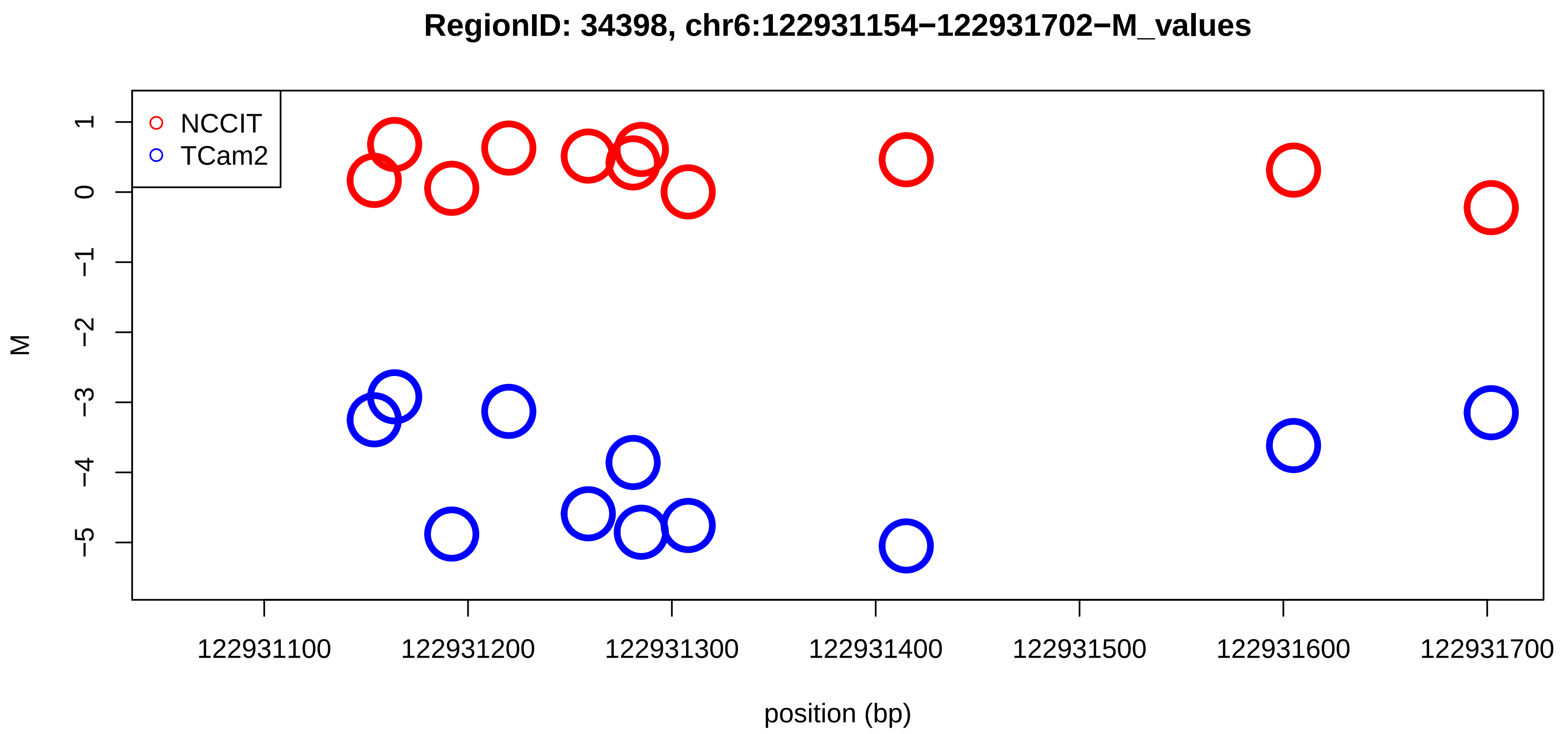
RegionID: 34398, chr6:122931154−122931702−M_values
NCCIT
TCam2
M
1
0
−1
−2
−3
−4
−5
122931100
122931200
122931300
122931400
122931500
122931600
122931700
position (bp)

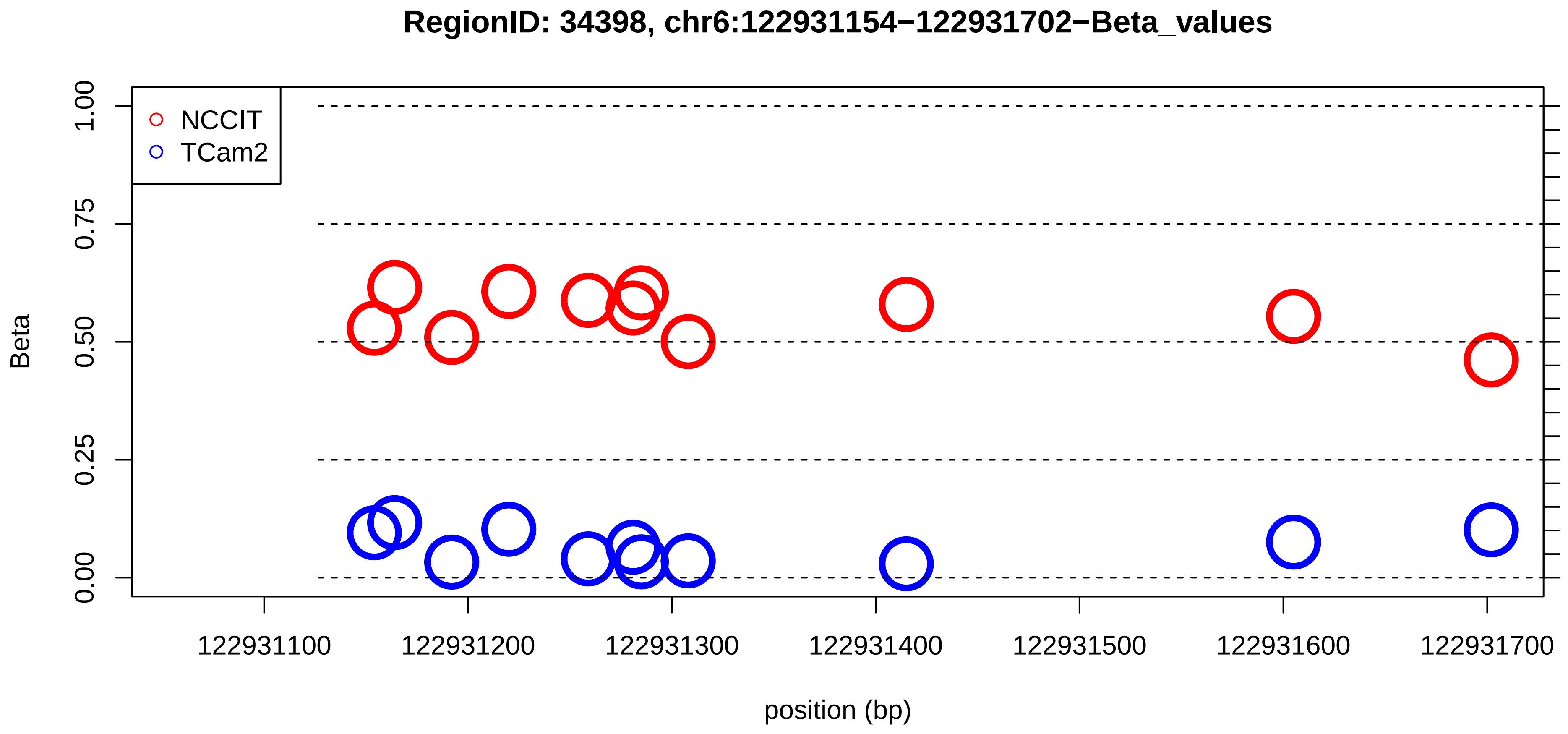
RegionID: 34398, chr6:122931154−122931702−Beta_values
NCCIT
TCam2
Beta
1.00
0.75
0.50
0.25
0.00
122931100
122931200
122931300
122931400
122931500
122931600
122931700
position (bp)

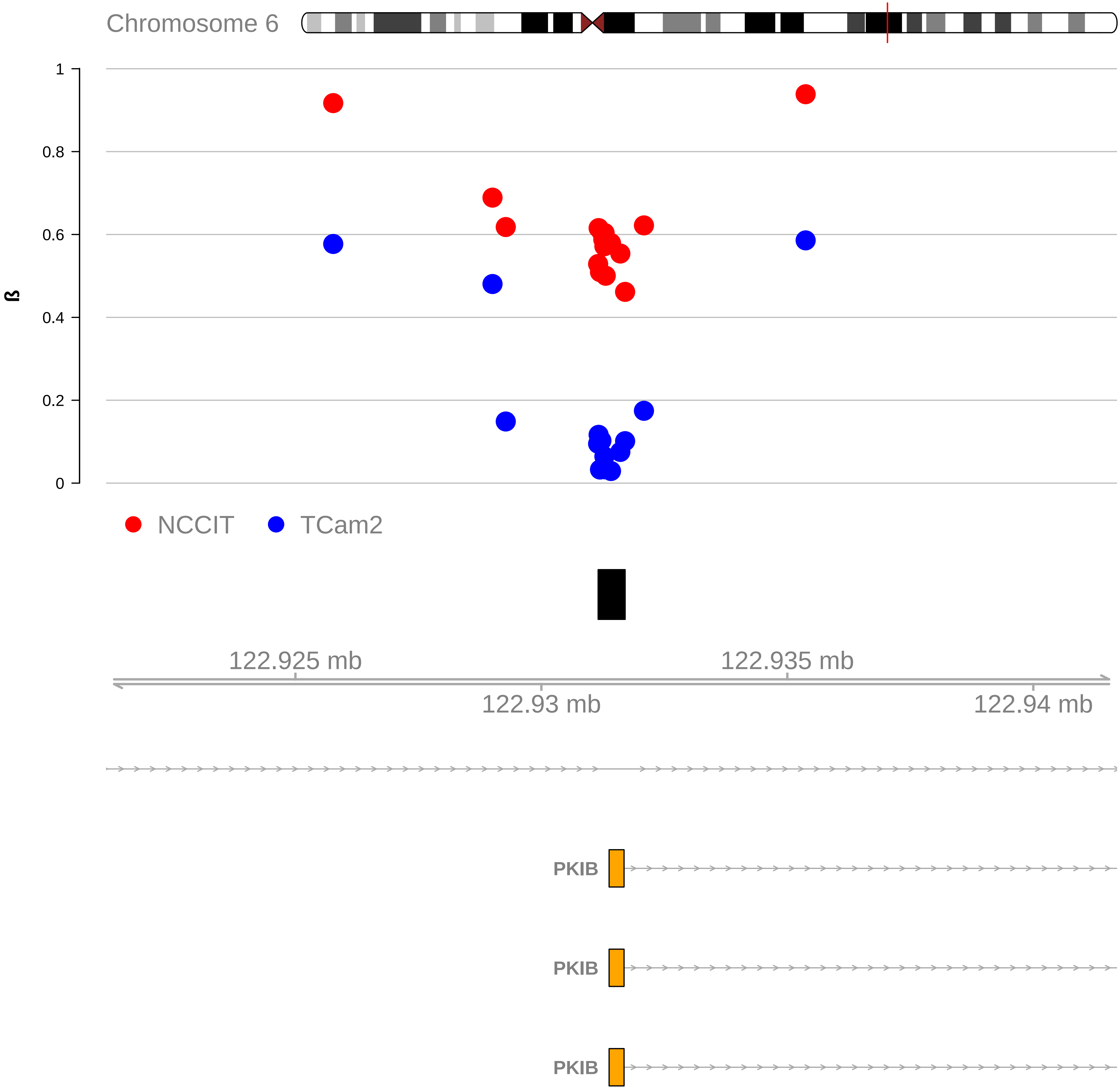

Chromosome 6
1
0.8
0.6
0.4
0.2
0
ß
NCCIT
TCam2
122.925 mb
122.935 mb
122.93 mb
122.94 mb
PKIB
PKIB
PKIB
